# Post-flowering Soil Waterlogging Curtails Grain Yield Formation by Restricting Assimilates Supplies to Developing Grains

**DOI:** 10.3389/fpls.2022.944308

**Published:** 2022-06-17

**Authors:** Shangyu Ma, Junyou Hou, Yanyan Wang, Mengchang Wang, Wenjing Zhang, Yonghui Fan, Zhenglai Huang

**Affiliations:** ^1^Key Laboratory of Wheat Biology and Genetic Improvement on Southern Yellow and Huai River Valley, Ministry of Agriculture and Rural Affairs, College of Agronomy, Anhui Agricultural University, Hefei, China; ^2^Jiangsu Collaborative Innovation Center for Modern Crop Production, Nanjing, China; ^3^Agricultural Park Management Center, Anhui Agricultural University, Hefei, China

**Keywords:** winter wheat, waterlogging, net photosynthetic rate, grain development, yield

## Abstract

Soil waterlogging is among the major factors limiting the grain yield of winter wheat crops in many parts of the world, including the middle and lower reaches of the Yangtze River China. In a field study, we investigated the relationship between leaf physiology and grain development under a varying duration of post-flowering waterlogging. A winter wheat cultivar Ningmai 13 was exposed to soil waterlogging for 0 (W0), 3 (W3), 6 (W6), and 9 d (W9) at anthesis. Increasing waterlogging duration significantly reduced flag leaf SPAD (soil plant analysis development) values and net photosynthetic rate (Pn). There was a linear reduction in flag leaf Pn and SPAD as plant growth progressed under all treatments; however, the speed of damage was greater in the waterlogged leaves. For example, compared with their respective control (W0), flag leaves of W9 treatment have experienced 46% more reduction in Pn at 21 d after anthesis (DAA) than at 7 DAA. Increasing waterlogging duration also induced oxidative damage in flag leaves, measured as malondialdehyde (MDA) contents. The capacity to overcome this oxidative damage was limited by the poor performance of antioxidant enzymes in wheat leaves. Inhibited leaf Pn and capacity to sustain assimilate synthesis under waterlogged environments reduced grain development. Compared with W0, W6 and W9 plants experienced a 20 and 22% reduction in thousand grain weight (TGW) in response to W6 and W9, respectively at 7 DAA and 11 and 19%, respectively at 28 DAA. Sustained waterlogging also significantly reduced grain number per spike and final grain yield. Averaged across two years of study, W9 plants produced 28% lesser final grain yield than W0 plants. Our study suggested that wheat crops are highly sensitive to soil waterlogging during reproductive and grain filling phases due to their poor capacity to recover from oxidative injury to photosynthesis. Management strategies such as planting time, fertilization and genotype selection should be considered for the areas experiencing frequent waterlogging problems.

## Introduction

Agricultural production is currently facing remarkable challenges in feeding a rapidly growing world population under diminishing natural resources (Godfray et al., 2011). This issue is further exacerbated by the global climate changes causing erratic rainfalls, prolonged drought, and intense heat waves during cropping seasons (Watanabe et al., 2018). For example, intermittent rains and subsequent soil waterlogging can affect approximately 10–15 million ha of global wheat-producing land, representing 15–20% of the cultivated area annually (Zhang et al., 2015). Wheat production in the middle and lower reaches of the Yangtze River, China accounts for approximately 26.3% of the total national planted area (National Bureau of Statistics of China, 2019). Wheat crop in this region is planted in saturated paddy fields, which results in a significant risk of intermittent soil waterlogging combined with rainfall without any seasonal distribution (Wu et al., 2018). Excessive precipitation can cause up to an 18.4% reduction in winter wheat yield in this region (Liu et al., 2022). Under the rapidly changing climatic extremes in recent years, the frequency of rainfall intensity and the possibility of soil waterlogging have significantly increased (Shao et al., 2013). For instance, waterlogging frequency in the spring season ranged between 7.5 and 33% across the meteorological station in this area (Wu et al., 2019).

The dominant monsoon climate in this region, especially from March to May, often carries abundant precipitation with an erratic spatial and temporal distribution (Ding et al., 2017). Consequently, varying degrees of waterlogging are jointly determined by local climate, terrain, soil properties, and underground water table level (Chen et al., 2018). This excessive precipitation usually coincides with sensitive growth phases of wheat crops, e.g., reproductive and grain filling phases. Considering the importance of the grain-filling period for final grain weight and yield formation (Fuentealba-Sandoval et al., 2020), waterlogging during this phase can significantly limit wheat productivity (Jiang et al., 2008). Understanding the physiological processes regulating grain development under soil waterlogging could assist in devising management strategies and protecting them from potential damage.

The negative effects of waterlogging on plants are initiated by soil oxygen deficiencies (Shaw et al., 2013). Under oxygen-deficient environments, plant roots switch from aerobic to anaerobic respiration, limiting energy production (Xuewen et al., 2014; Zhou et al., 2020). Inhibited root functioning, in turn, can impact leaf physiology, i.e., stomatal conductance (Sairam et al., 2008), carbon assimilation (Malik et al., 2001), and overall plant growth. Wheat leaves are the primary photosynthetic organ, with the flag leaf alone contributing to approximately 41–43% of total plant assimilation (Ross et al., 1984). Inhibited oxygen supplied under waterlogged soils can significantly damage chlorophyll and photosynthetic rate (Najeeb et al., 2015a,b). The most evident characteristic of wheat leaf senescence is the gradual decomposition of chlorophyll, which is manifested as the loss of green, yellow, and dry leaves (Lv et al., 2020). Significant damage to chloroplast structure and carbon assimilation process in wheat has been recorded under short-term (5 d) waterlogging (Zheng et al., 2009).

Comparing the performance of wheat genotypes in response to soil waterlogging (21 d) during different developmental phases, i.e., jointing, flowering, and grain filling, Araki et al. (2012) suggested that wheat crops are susceptible to post-flowering waterlogging. They also proposed that post-flowering waterlogging can arrest grain development by accelerating leaf senescence. This accelerated leaf senescence could be associated with unregulated reactive oxygen species (ROS) generation in oxygen-deficient cells of waterlogged plants, damaging lipid membranes and chloroplast structure (Blokhina et al., 2001). Zheng et al. suggested that soil waterlogging induces lipid membrane peroxidation, inhibiting light reaction systems and ATP generation in wheat leaves (Zheng et al., 2009). Some stressed plants can minimize this damage by modifying their antioxidant enzyme activities and detoxifying ROS (Sarwar et al., 2017). For instance, Spanic et al. (2020) recorded a significant change in the activities of antioxidant enzymes such as ascorbate peroxidase (APX), guaiacol peroxidase (GPOD), polyphenol oxidase (PPO), and catalase (CAT) in the flag leaves of waterlogged wheat.

Sustained carbohydrate and nutrient supplies are essential for wheat grains during the critical phases of grain development (Ullah and Chenu, 2019). Soil waterlogging can impact these supplies by impairing leaf physiological functioning. In this study, a winter wheat variety, ‘Ningmai 13’, is exposed to different durations post-flowering waterlogging to understand the relationship between leaf physiology and grain development. The information will be used for developing management techniques for improving wheat performance under waterlogged environments.

## Materials and Methods

### Growing Conditions

**Figure 1:**
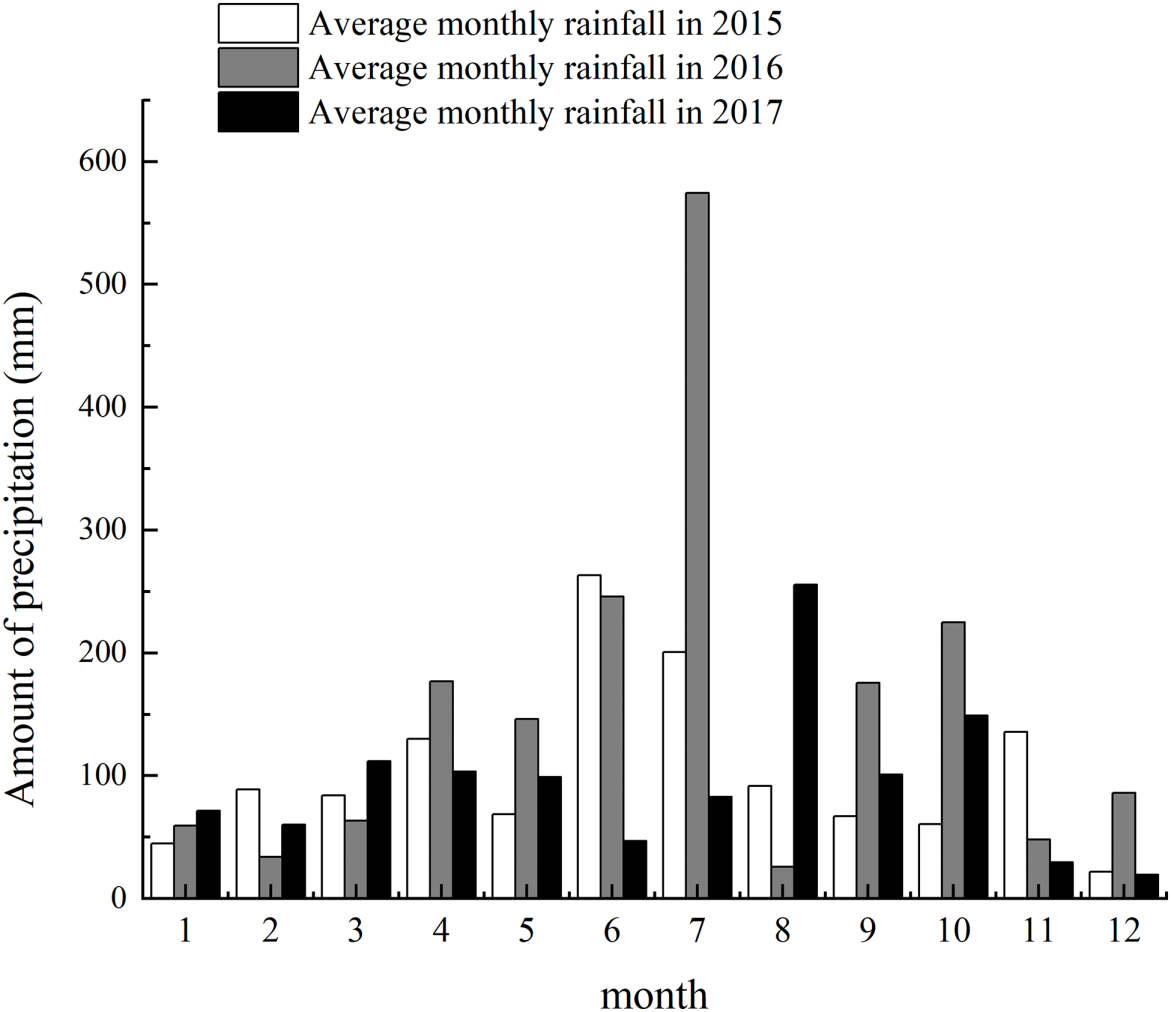
Precipitation of the test field in two planting seasons.

Field experiments were conducted at the experimental station of Anhui Agricultural University (117.01′ E, 30.57′ N) in Lujiang County, Hefei City, Anhui Province, during 2015–16 and 2016–17. The region has a subtropical humid monsoon climate with an average annual precipitation, sunshine duration, and frost-free period of 1188.1 mm, 2209.6 h, and 238 d, respectively. The monthly precipitation during growing seasons is shown in Figure 1.

### Experimental Design

Each experimental plot (2.4 m × 5.0 m) consisted of 13 rows with 20 cm spacing to achieve a target population of 300 plants per square meter. The treatments were replicated thrice in randomized complete block designs. The plot edges were insulated from each other with a plastic frame made of polyvinyl chloride. The plastic frame was buried 40 cm deep and extended 20 cm on the ground. The treatment plots were artificially waterlogged when 50% of the plants in the field passed flowering [Zadoks decimal growth stage (Zadoks et al., 1974), Z65]. The water layer was kept 2 cm above the ground for 0, 3, 6, and 9 d, which were recorded as W0, W3, W6, and W9, respectively. At the end of each waterlogging period, water was discharged from the plots, and crops were irrigated normally until maturity.

### Crop Management

A major winter wheat cultivar in the middle and lower reaches of the Yangtze River China ‘Ningmai 13’ was used as a test cultivar. The seeds were sown on 8 November 2015 and 11 November 2016.

All plots were supplied with 225 kg N ha^−1^, 75 kg P_2_O_5_ ha^−1^, and 150 kg K_2_O ha^−1^. All P and K fertilizers and 70% of N fertilizers were applied before sowing, and the remaining N fertilizer was top-dressed at jointing. Wheat plants were harvested on 24 May 2016 and 27 May 2017.

### Measurements

#### SPAD Value and Pn of Flag Leaves

Leaf physiological data were recorded from all the plots at 7, 14, and 21 DAA. For this purpose, five tillers per replicate were tagged at flowering, and the same flag leaves were used for measuring SPAD and Pn.

The SPAD value was measured non-destructively using SPAD 502 Meter (Soil Plant Analysis Development, Minolta, Japan). For each measurement, five SPAD readings were obtained and averaged.

The Pn values were measured using a portable photosynthesis system (LI-6400, LI-Cor, United States) at a CO_2_ concentration of about 385 mol, and the reading of the light source was 1,200 mol·m^−2^·s^−1^. All measurements were made between 9:00 and 11:00 on days with full sunlight (Mu et al., 2010).

#### Malondialdehyde Content and Cat and Sod Activities

For the destructive measurement, 15 tillers per replicate were tagged at flowering. Flag leaf samples were collected from the tagged tiller (5 tillers each time) at 7, 14, and 21 d after flowering. The sampled leaves were pooled and stored in liquid nitrogen until analysis. Catalase (CAT) and superoxide dismutase (SOD) activities and MDA content were determined according to Pang et al. (2002) and Spanic et al. (2020).

#### Grain Weight and Yield

For grain yield measurements, emerging flowering spikes were tagged on the same day. Twenty tagged spikes from each experimental plot were sampled at 7-day intervals from the beginning of anthesis to maturity. The grain filling rate was estimated from the accumulation of dry grain weight. At each sampling date, grains were separated from the glumes and dried at 70°C until a constant weight was reached. The total number of grains was determined, and their dry weight was recorded.

The number of spikes and grains per spike were investigated at the mature stage, and each plot was separately threshed, weighed, and converted into the yield when the water content was 13%.

### Statistical Analysis

Data were subjected to ANOVA (SSPS for Windows, version 22.0). The ANOVA used a level of significance of *α* = 0.05 to identify significant differences among treatments. Multiple comparisons were made using the least significant difference test with *α* = 0.05 to determine significant differences among treatments. The Origin9.1 (Origin Lab, Northampton, MA, United States) was used to show differences in SPAD values, Pn values, MDA content, SOD and CAT activities and TGW.

## Results

### SPAD Values of Flag Leaves Under Different Waterlogging Durations

**Figure 2:**
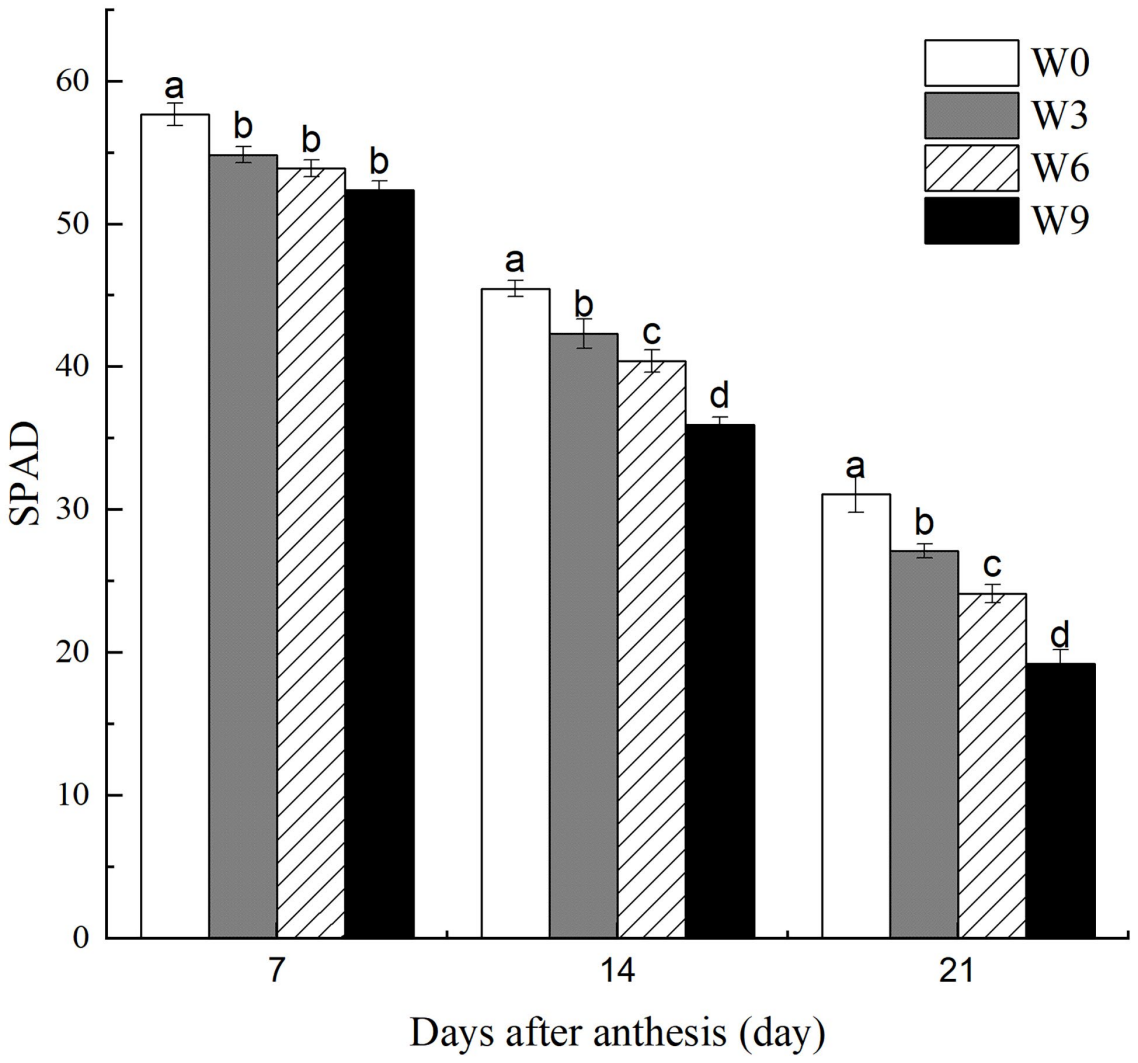
SPAD values of wheat flag leaves under varying waterlogging treatments. Data were collected at 7, 14, and 21 days after anthesis. W0 = control, W3 = 3 days of waterlogging, W6 = 6 days of waterlogging, W9 = 9 days of waterlogging. Vertical bars represent the mean of each treatment (six replicates across two years) ± SE (standard errors). Means carrying the same letter are not significantly different at the 5% level.

Increasing waterlogging duration from 3 to 9 d significantly reduced leaf SPAD values at 14 and 21 DAA, although there was no significant impact of waterlogging duration on these traits at 7 DAA (Figure 2). Further, SPAD values for each treatment were significantly reduced as the plant growth progressed with each subsequent measurement (i.e., 7, 14, and 21 DAA). For example, compared with their respective controls (W0), W3, W6, and W9 plants had 4.9, 6.6, and 9.2% (averaged across tested years) lower leaf SPAD, respectively, at 7 DAA, but these reductions were 12.8, 22.3, and 38.2%, respectively, at 21 DAA.

### Pn of Flag Leaves Under Different Waterlogging Durations

**Figure 3:**
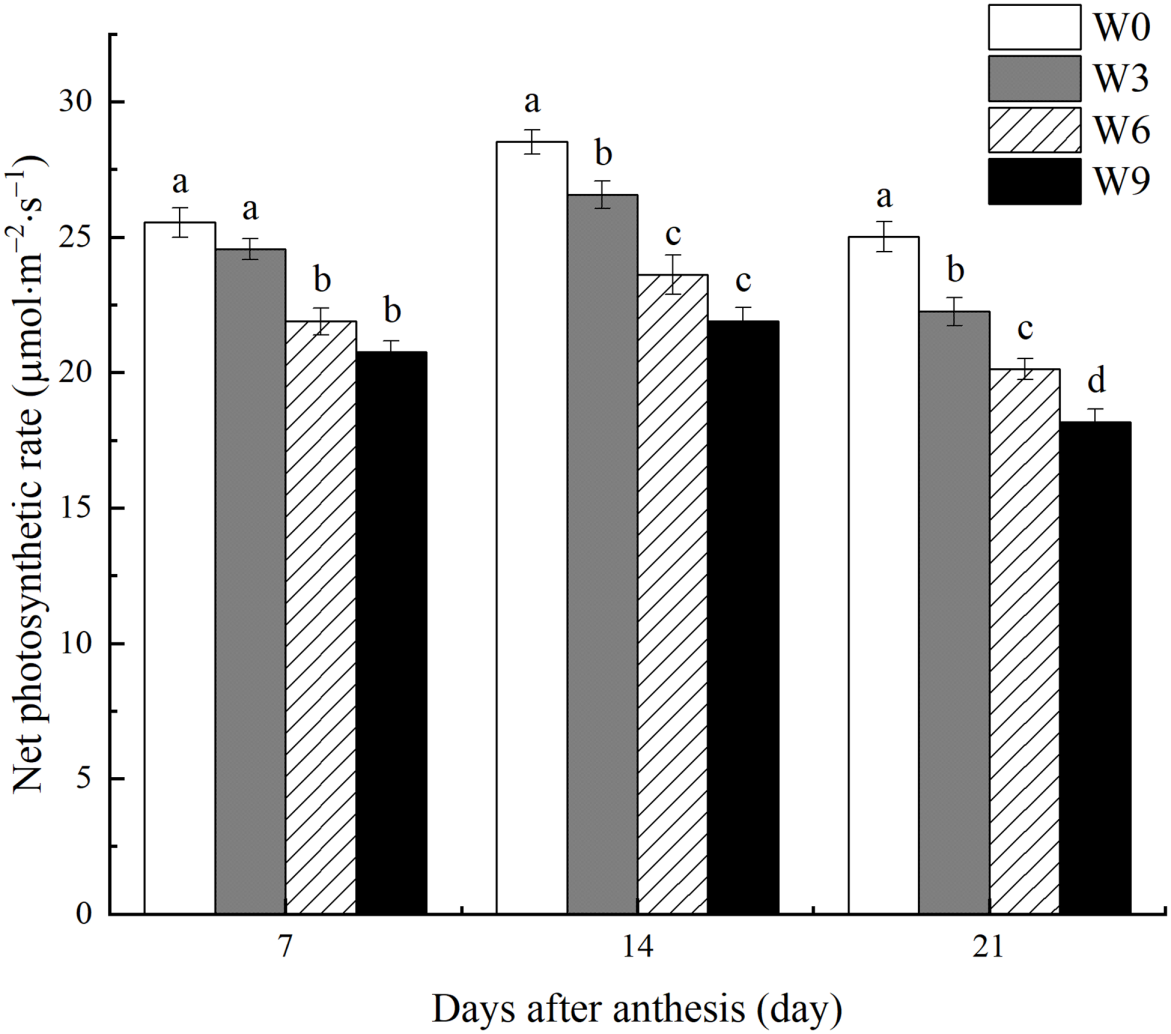
Net photosynthetic rate of flag leaves under varying waterlogging durations. Data were collected at 7, 14, and 21 days after anthesis. W0 = control, W3 = 3 days of waterlogging, W6 = 6 days of waterlogging, W9 = 9 days of waterlogging. Vertical bars represent the mean of each treatment (six replicates across two years) ± SE (standard errors). Means carrying the same letter are not significantly different at the 5% level.

Short-term waterlogging (W3) had no significant effect on the net photosynthetic rate (Pn) of flag leaves at 7 DAA, but it significantly reduced leaf Pn at 14 and 21 DAA (Figure 3). W6 and W9 significantly reduced Pn during all the tested developmental stages. Averaged across two years, W6 and W9 reduced leaf Pn by 14.3 and 18.7%, respectively at 7 DAA, and 17.2 and 23.2%, respectively at 14 DAA, and 19.5 and 27.4%, respectively at 21 DAA. Furthermore, all plants had a maximum Pn at 14 DAA and then reduced at 21 DAA.

### SOD Activity in Flag Leaves Under Different Waterlogging Durations

**Figure 4:**
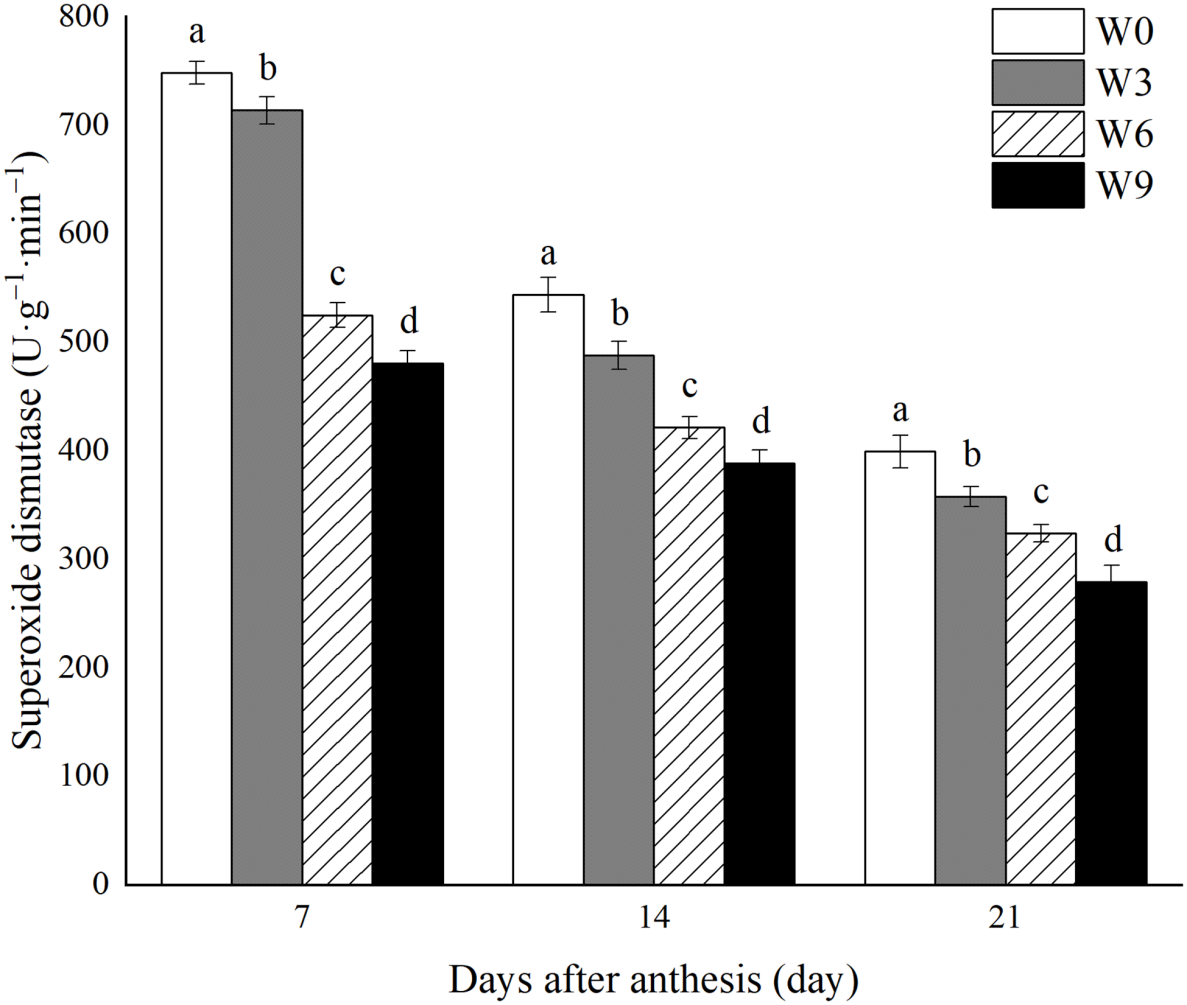
SOD activity in wheat flag leaves under varying waterlogging durations. Data were collected at 7, 14, and 21 days after anthesis. W0 = control, W3 = 3 days of waterlogging, W6 = 6 days of waterlogging, W9 = 9 days of waterlogging. Vertical bars represent the mean of each treatment (six replicates across two years) ± SE (standard errors). Means carrying the same letter are not significantly different at the 5% level.

Increasing waterlogging duration significantly reduced SOD activity in flag leaves of wheat during all the tested developmental stages (Figure 4). Also, SOD activity under each treatment level gradually decreased with the wheat growth. Compared with their respective control (W0) and averaged across the tested years, W3, W6 and W9 had 4.6, 29.8 and 35.7% lower SOD activity, respectively, at 7 DAA, 10.2, 22.5 and 28.5%, respectively, at 14 DAA and 10.4, 18.9 and 30.1%, respectively at 21 DAA (Figure 4).

### CAT Activity in Flag Leaves Under Different Waterlogging Durations

**Figure 5:**
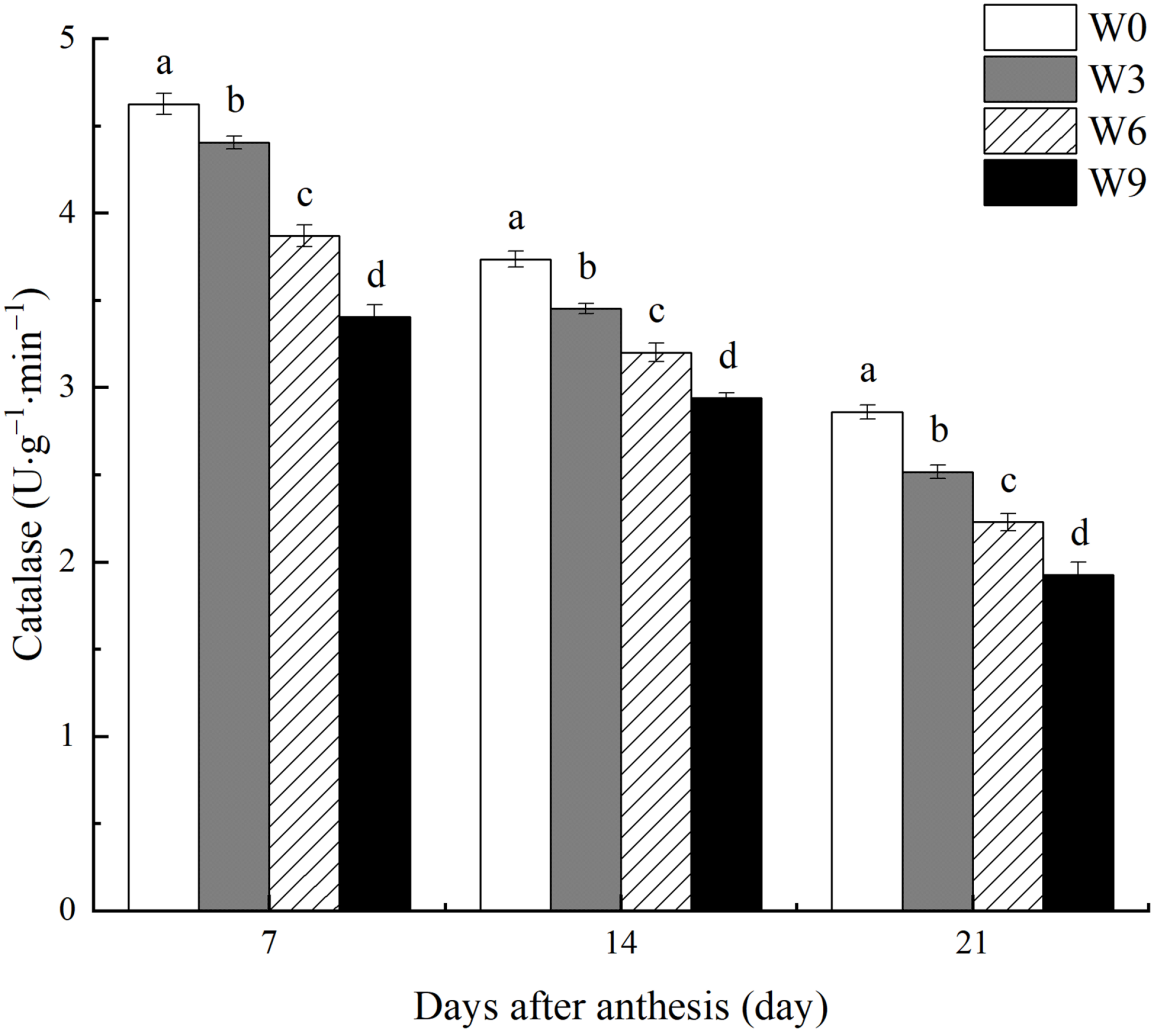
CAT activity in wheat flag leaves under varying waterlogging durations. Data were collected at 7, 14, and 21 days after anthesis. W0 = control, W3 = 3 days of waterlogging, W6 = 6 days of waterlogging, W9 = 9 days of waterlogging. Vertical bars represent the mean of each treatment (six replicates across two years) ± SE (standard errors). Means carrying the same letter are not significantly different at the 5% level.

During the two tested growing seasons, increasing waterlogging duration significantly reduced CAT activity in flag leaves during all developmental phases (Figure 5). CAT activity in flag leaves decreased as plant growth progressed, with a further intensification of the impact of W3 on CAT, although W6 and W9 impact did not change much. Compared with their respective control (W0) and averaged across the tested years, W3, W6, and W9 reduced CAT activity in flag leaves by 4.8, 16.4, and 26.4%, respectively at 7 DAA, by 7.6, 14.3, and 21.3% respectively, at 14 DAA, and by 12.0, 22.1, and 32.7%, respectively at 21 DAA.

### MDA Content in Flag Leaves Under Different Waterlogging Durations

**Figure 6:**
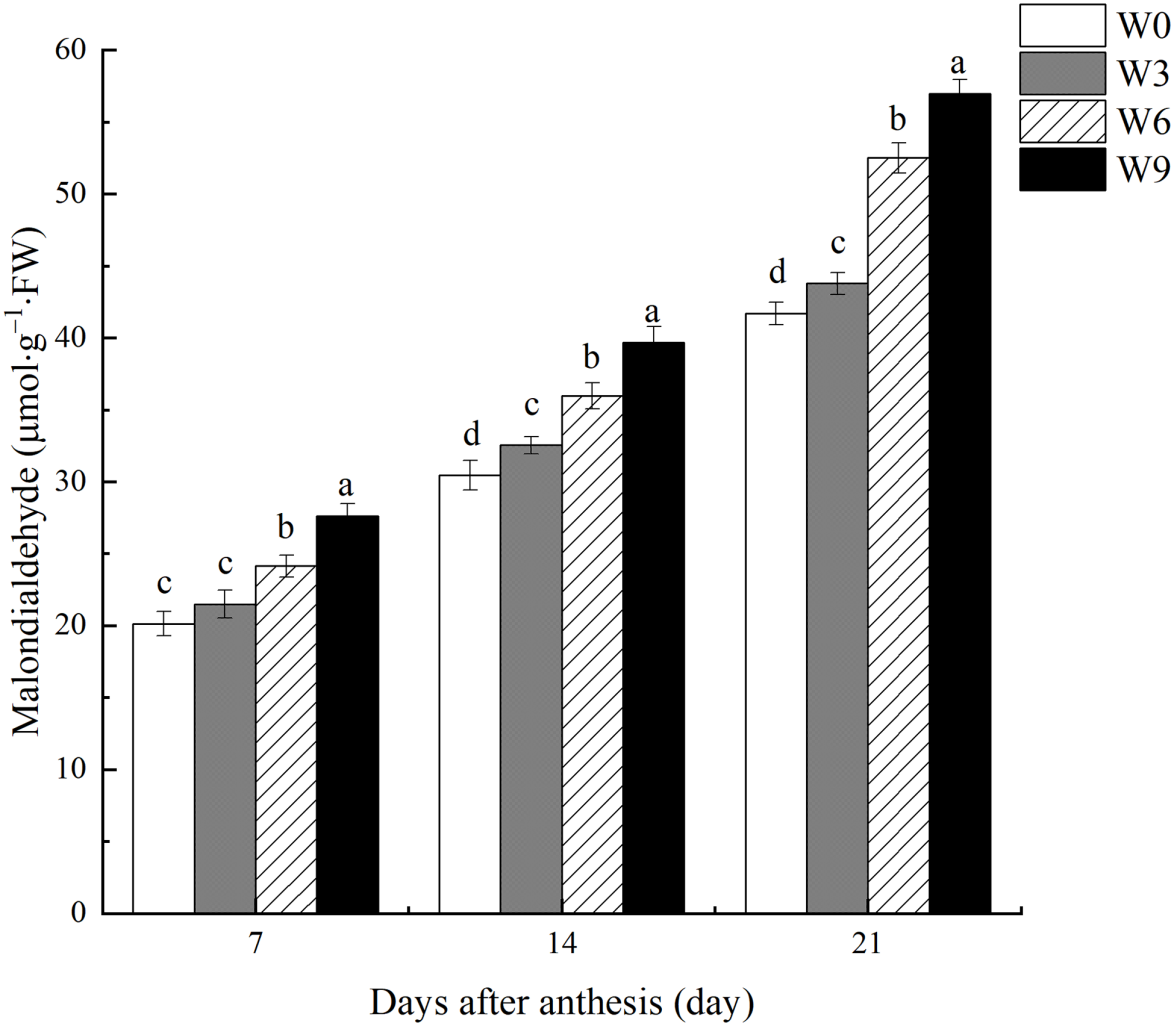
MDA contents in wheat flag leaves under varying waterlogging durations. Data were collected at 7, 14, and 21 days after anthesis. W0 = control, W3 = 3 days of waterlogging, W6 = 6 days of waterlogging, W9 = 9 days of waterlogging. Vertical bars represent the mean of each treatment (six replicates across two years) ± SE (standard errors). Means carrying the same letter are not significantly different at the 5% level.

Damage to flag leaf lipid membrane calculated in terms of MDA contents increased as the plant growth progressed with each subsequent measurement (Figure 6). Increasing waterlogging duration significantly increased MDA contents, with an accelerated increase under W6 and W9 at 21 DAA. For example, compared with W0 and averaged across two years, W6 and W9 increased MDA contents by 19.95 and 28.40%, respectively, at 7 DAA, but this increase was intensified to 25.9, and 30.2%, respectively, at 21 DAA.

### TGW Under Different Waterlogging Durations

**Figure 7:**
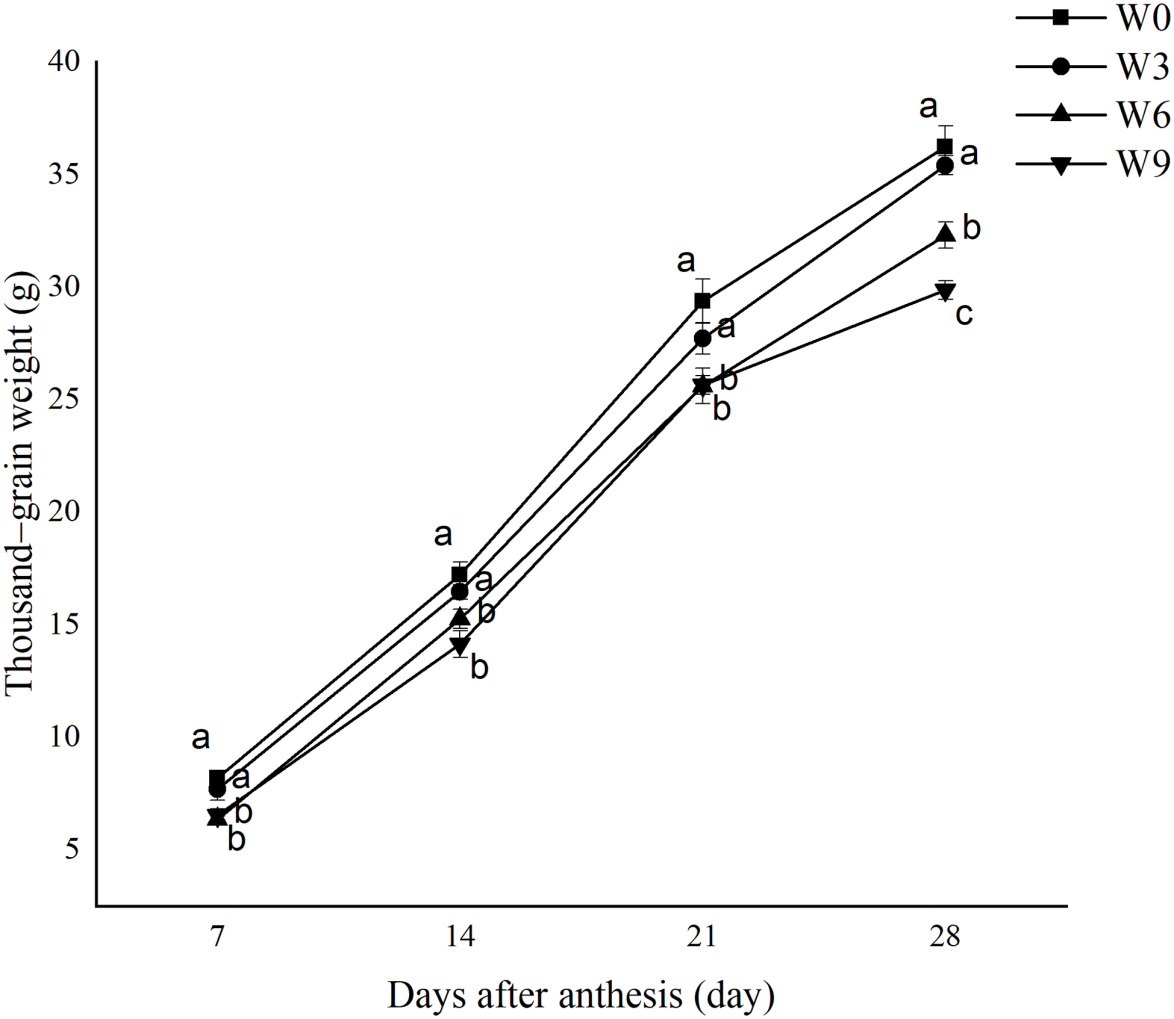
Thousand-grain weight under different waterlogging durations. Data were collected at 7, 14, 21, and 28 days after anthesis. W0 = control, W3 = 3 days of waterlogging, W6 = 6 days of waterlogging, W9 = 9 days of waterlogging. The values are the mean of each treatment (six replicates across two years) ± SE (standard errors). Means carrying the same letter are not significantly different at the 5% level.

During all the tested developmental stages, W3 had no significant effect on TGW, but W6 and W9 significantly reduced TGW during all developmental phases. This waterlogging-induced reduction in TGW was intensified as plant growth progressed. W6 and W9 had no significant effect during earlier and middle stages of grain development (i.e., 7, 14, and 21 DAA); however, the TGW of W9 was significantly lower than that of W6 at 28 DAA (Figure 7). Compared with W0 and averaged across two years, W3, W6, and W9 reduced TGW by 5.61, 12.83, and 12.72%, respectively, at 21 DAA and 2.25, 10.83, and 17.54%, respectively at 28 DAA.

### Principal Component Analysis

**Figure 8:**
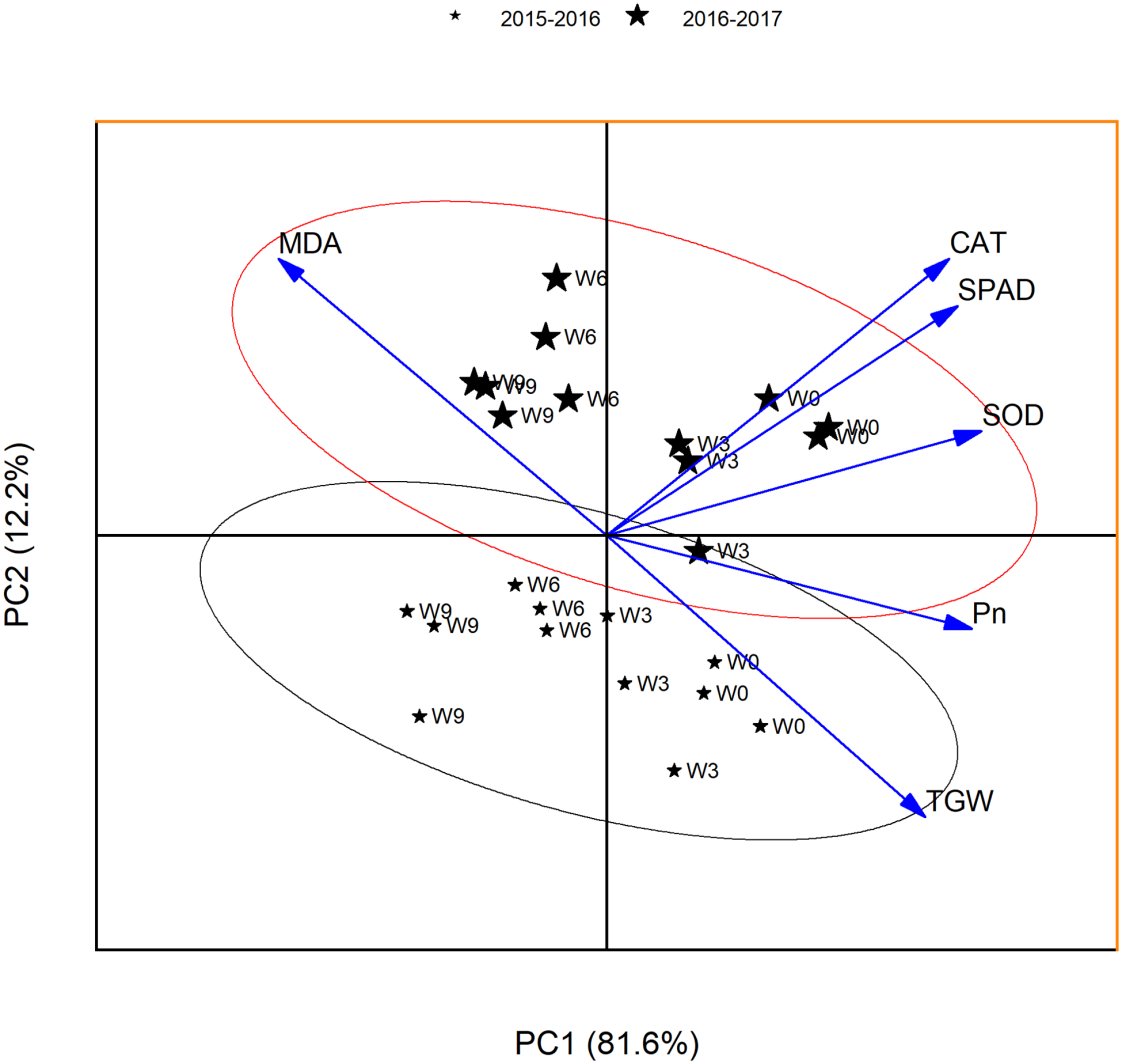
PCA for the yield-related traits at 21 DAA. W0 = control, W3 = 3 days of waterlogging, W6 = 6 days of waterlogging, W9 = 9 days of waterlogging.

To find the relationships between the analyzed parameters, a principal component analysis (PCA) was performed. PCA analysis allows for the determination of variables that exert the greatest impact on the appearance of individual components. The presented PCA facilitates the interpretation of the impact of Pn value, MDA content, and antioxidant enzyme activities on TGW. Two main components derived from the calculations were selected; they allowed for the explanation of 93.8% of the total variance of analyzed variables. The first main component contains 81.6% of the information about the tested samples represented by variables, while the second main component contains 12.2% of the information (Figure 8).

Coefficients of PC1 (eigenvectors) leaf Pn, SPAD, and antioxidant enzymes were positive, while MDA was negative. Significantly higher values of eigenvectors for SPAD and MDA indicate superior leaf SPAD and lower MDA contents are a good indicator of plant performance under waterlogging. This separated the plants on the basis of waterlogging treatment in the same direction. W0 plants were grouped together (the top right hand side of the axis), indicating a strong association of TGW with Pn and other leaf physiological traits. With the increasing waterlogging duration, the plants were grouped to the left hand side of the axis, indicating higher MDA level could reduce grain size. The eigenvectors values of PCA indicated that PC2 was an index of the subtle differences in Pn and MDA contents, and thus could explain the variable grouping of plants based on the waterlogging duration.

### Yield and Yield Components Under Different Waterlogging Durations

Waterlogging treatments had no significant effect on the total number of spikes in this study, but other grain yield components such as kernel numbers, TGW, and total grain yield all were significantly affected by W6 and W9 treatment (Table 1). W3 had no significant effect on spikes, kernel numbers, TGW, and the total grain yield. W9 plants experienced a significant reduction in TGW and total grain yield compared with W6 plants. Averaged across two years, compared with W0, kernel numbers, TGW, and grain yield of W6 and W9 were reduced by 8.0 and 9.9%, 11.0 and 17.9%, 18.8 and 28.8%, respectively.

**Table 1:** Wheat yield and its components under different waterlogging treatments at crop maturity.

Growing season	Treatment	Spikes (10^4^ per ha)	Kernel numbers per spike	Thousand grain weight (g)	Grain yield (kg ha^−1^)
2015–2016	W0	522.3a	34.9a	36.9a	6235.6a
	W3	510.6a	33.8a	36.5a	6005.4a
	W6	515.7a	32.0b	33.2b	5049.0b
	W9	509.6a	30.8b	30.4c	4344.2c
2016–2017	W0	560.2a	32.7a	37.2a	6201.2a
	W3	555.2a	31.7a	35.3a	5939.4a
	W6	555.3a	30.1b	32.8b	5012.7b
	W9	542.4a	30.0b	30.4c	4483.6c
Averaged across two years	W0	541.3a	33.8a	37.1a	6218.4a
	W3	532.9a	32.8a	35.9a	5972.4a
	W6	535.5a	31.1b	33.0b	5030.8b
	W9	526.0a	30.4b	30.4c	4413.9c

Different lowercase letters in the same column indicate significant differences among treatments (p < 0.05).

## Discussion

### Inhibited Assimilates Supply Reduces Grain Development

In this study, increasing waterlogging duration significantly reduced leaf greenness (SPAD value), and the damage accelerated as the plant progressed. Flag leaf SPAD value is considered a good indicator of plant health (Mielke et al., 2010). Earlier studies suggested that sustained waterlogging reduced leaf SPAD limiting N uptake (Wu et al., 2014). Inhibited oxygen supplies in the waterlogged soils can also arrest chlorophyll synthesis and accelerate senescence (Zheng et al., 2009). In this study, premature leaf senescence, particularly under long-term waterlogging (W6 and W9), could have affected the length of the functional grain filling period and, thus, finally, grain yield.

Leaf chlorophyll is a site for photosynthesis and assimilates production. In plants, the assimilation process is strongly associated with leaf age, leaf chlorophyll content, and the activity of enzymes (Thomas and Ougham, 2014; Liu et al., 2015; Bhusal et al., 2020). Developing grains require continuous assimilate supply which is either met through current (photosynthesis) or stem stored assimilates in wheat (Ullah and Chenu, 2019). This assimilates supply during mid to late grain filling phases of grain development in wheat is crucial for sustaining grain size (Hou et al., 2020). In the current study, waterlogging inhibited photosynthesis, curtailing a balance between assimilate supplies and deposition in grain. This damage was intensified with plant growth and increasing waterlogging duration, i.e., W9 plants had 27.4% lower Pn than W0 at 21 DAA. This inhibited assimilates supplies, directly affected the grain-filling process, and reduced grain size (TGW).

### Oxidative Stress and Lipid Membrane Damage

In this study, increasing waterlogging duration significantly damaged the lipid membrane of flag leaf tissues in wheat. Excessive ROS and oxidative damage are commonly observed phenomena in stressed plants. These ROS can harm cellular membrane permeability and thus inhibit plant growth, development, and final yield (Jing et al., 2020). Under waterlogged or hypoxic conditions, ROS generation is associated with changes in the redox status of plant species (Ricard et al., 1994). No immediate effect of short-term waterlogging (W3) on MDA contents indicates that wheat plants could temporarily sustain energy production through glycolysis and ethanol fermentation. However, the damage was further exacerbated as plant growth progressed (Figure 6), suggesting that wheat plants were unable to recover from post-waterlogging oxidative stress. Plants may overcome this oxidative damage by activating their antioxidant enzyme system (El Sabagh et al., 2019; Yu et al., 2020). In this study, a significant reduction in antioxidant enzymes, i.e., SOD and CAT, in response to soil waterlogging suggested limited capacity of the tested wheat genotype to detoxify unregulated ROS.

Unregulated cellular ROS can increase the peroxide level (Yusuf et al., 2008), damaging organelles, such as chloroplasts, mitochondria, plasma membrane, exosomes, and nucleoli. ROS can induce the production of MDA in biofilm-damaged cells, and the content of MDA can directly reflect the aging of the organism when damaged by water streaming (Sandalio et al., 2013). In this study, accelerated chlorophyll loss in wheat flag leaves could result from oxidative damage to the chloroplast lipid membrane (Dat et al., 2003).

### Yield Loss Caused by Restricted Grain Development

In this study, waterlogging significantly reduced grain yield components, including TGW and grain number per spike. This yield loss was strongly associated with waterlogging duration, i.e., grain yield loss increased from 3.7 to 28.8% by increasing waterlogging duration from 3 to 9 d. Significant grain yield loss, i.e., up to 40%, has been recorded in wheat crops in response to sustained waterlogging (Ding et al., 2020). Soil waterlogging can significantly accelerate ethylene biosynthesis in plant tissues (Najeeb et al., 2015a), a hormone responsible for grain loss in wheat (Hays et al., 2007). Final grain size in wheat depends on multiple factors such as grain filling duration, assimilate synthesis and transport, and starch deposition in grains (Jenner et al., 1991). In this study, waterlogging accelerated leaf senescence and inhibited photosynthesis, reducing assimilates supplies to the developing wheat grains. Despite a significant reduction in flag leaf Pn in response to W3 during later developmental phases, final TGW was not significantly affected by the short-term waterlogging. This indicated that continuous carbohydrates supply from current sources (photosynthesis) during the early phases of grain development is critical for sustaining grain growth in wheat. However, developing grains may become more dependent on assimilates supplies from the stored carbohydrate during later phases of development (Pheloung and Siddique, 1991). Girousse et al. (2021) suggested that damage to developing grain during the cellular division phase (0–7 DAA) is irreversible.

## Conclusion

In this study, grain yield loss increased from 3.7 to 28.8% by increasing waterlogging duration from 3 to 9 d. This grain yield loss was due to oxidative stress and lipid membrane damage in flag leaves, which accelerated leaf senescence and inhibited photosynthesis, reducing assimilates supplies to the developing wheat grains. This study found that short-term waterlogging (W3) reduced current sources (photosynthesis) during later phases of grain development only (14 and 21 d after anthesis), resulting in no significant reduction in TGW. However, the impact of W6 and W9 significant on Pn during the first week of waterlogging and thus these treatments reduced TGW. Our study suggested that sustain carbohydrates supplies are critical for developing wheat grains during early phases development. Crop management techniques should be adjusted to sustain assimilates supplies during this critical period, particularly under stressed environments.

## Data Availability Statement

The original contributions presented in the study are included in the article/supplementary material, further inquiries can be directed to the corresponding author.

## Author Contributions

SM and ZH contributed to conception and design of the study. JH organized the database. SM, JH, and YW performed the statistical analysis. SM wrote the first draft of the manuscript. All authors contributed to the article and approved the submitted version.

## Funding

This research was supported by the National Natural Science Foundation of China (31801287), the grants from the National Key Research and Development Program of China (2016YFD0300405 and 2017YFD0301305), and the Project of China Scholarship Council (201908775002).

## Conflict of Interest

The authors declare that the research was conducted in the absence of any commercial or financial relationships that could be construed as a potential conflict of interest.

## Publisher’s Note

All claims expressed in this article are solely those of the authors and do not necessarily represent those of their affiliated organizations, or those of the publisher, the editors and the reviewers. Any product that may be evaluated in this article, or claim that may be made by its manufacturer, is not guaranteed or endorsed by the publisher.
